# Effects of Physical and Mental Fatigue on Postural Sway and Cortical Activity in Healthy Young Adults

**DOI:** 10.3389/fnhum.2022.871930

**Published:** 2022-06-14

**Authors:** Arnd Gebel, Aglaja Busch, Christine Stelzel, Tibor Hortobágyi, Urs Granacher

**Affiliations:** ^1^Division of Training and Movement Sciences, Research Focus Cognition Sciences, University of Potsdam, Potsdam, Germany; ^2^University Outpatient Clinic, Sports Medicine and Sports Orthopedics, University of Potsdam, Potsdam, Germany; ^3^Physiotherapy, Department of Health Professions, Bern University of Applied Sciences, Bern, Switzerland; ^4^General Psychology and Neurocognitive Psychology, Berlin, Germany; ^5^University Medical Center Groningen, Center for Human Movement Sciences, University of Groningen, Groningen, Netherlands; ^6^Somogy County Kaposi Mór Teaching Hospital, Kaposvár, Hungary; ^7^Department of Sport Biology, Institute of Sport Science and Physical Education, University of Pécs, Pécs, Hungary; ^8^Department of Kinesiology, University of Physical Education, Budapest, Hungary

**Keywords:** balance, cognitive/muscular fatigue, EEG, theta, alpha-2

## Abstract

Physical fatigue (PF) negatively affects postural control, resulting in impaired balance performance in young and older adults. Similar effects on postural control can be observed for mental fatigue (MF) mainly in older adults. Controversial results exist for young adults. There is a void in the literature on the effects of fatigue on balance and cortical activity. Therefore, this study aimed to examine the acute effects of PF and MF on postural sway and cortical activity. Fifteen healthy young adults aged 28 ± 3 years participated in this study. MF and PF protocols comprising of an all-out repeated sit-to-stand task and a computer-based attention network test, respectively, were applied in random order. Pre and post fatigue, cortical activity and postural sway (i.e., center of pressure displacements [CoP_d_], velocity [CoP_v_], and CoP variability [CV CoP_d_, CV CoP_v_]) were tested during a challenging bipedal balance board task. Absolute spectral power was calculated for theta (4–7.5 Hz), alpha-2 (10.5–12.5 Hz), beta-1 (13–18 Hz), and beta-2 (18.5–25 Hz) in frontal, central, and parietal regions of interest (ROI) and baseline-normalized. Inference statistics revealed a significant time-by-fatigue interaction for CoP_d_ (*p* = 0.009, *d* = 0.39, Δ 9.2%) and CoP_v_ (*p* = 0.009, *d* = 0.36, Δ 9.2%), and a significant main effect of time for CoP variability (CV CoP_d_: *p* = 0.001, *d* = 0.84; CV CoP_v_: *p* = 0.05, *d* = 0.62). *Post hoc* analyses showed a significant increase in CoP_d_ (*p* = 0.002, *d* = 1.03) and CoP_v_ (*p* = 0.003, *d* = 1.03) following PF but not MF. For cortical activity, a significant time-by-fatigue interaction was found for relative alpha-2 power in parietal (*p* < 0.001, *d* = 0.06) areas. *Post hoc* tests indicated larger alpha-2 power increases after PF (*p* < 0.001, *d* = 1.69, Δ 3.9%) compared to MF (*p* = 0.001, *d* = 1.03, Δ 2.5%). In addition, changes in parietal alpha-2 power and measures of postural sway did not correlate significantly, irrespective of the applied fatigue protocol. No significant changes were found for the other frequency bands, irrespective of the fatigue protocol and ROI under investigation. Thus, the applied PF protocol resulted in increased postural sway (CoP_d_ and CoP_v_) and CoP variability accompanied by enhanced alpha-2 power in the parietal ROI while MF led to increased CoP variability and alpha-2 power in our sample of young adults. Potential underlying cortical mechanisms responsible for the greater increase in parietal alpha-2 power after PF were discussed but could not be clearly identified as cause. Therefore, further future research is needed to decipher alternative interpretations.

## Introduction

Postural balance is an essential prerequisite to successfully perform everyday and sports-related activities (Horak, 2006; Kiers et al., 2013). For decades, it has been speculated that balance is controlled predominantly by spinal and subcortical systems (brain stem, basal ganglia, and cerebellum) (Dietz et al., 1991; Shumway-Cook and Woollacott, 2012). However, evidence from numerous neuroimaging studies supports the hypothesis that the maintenance of posture underlies complex interactions within specific regions of the somatosensory system (Jacobs and Horak, 2007). In fact, the performance of balance exercises with increasing task difficulty resulted in increases in theta band activity of fronto-central areas and decreases in alpha-2 frequency band power of centro-parietal regions using electroencephalography (EEG) (Hülsdünker et al., 2015a; Gebel et al., 2020). Solis-Escalante et al. (2019) and Varghese et al. (2019) reported that the application of postural perturbations resulted in multifocal transient changes in the frequency band power which could be indicative of cortical network activity involved in postural control. These study findings indicate modifications in balance performance are accompanied by changes in cortical activity (for a review see Wittenberg et al., 2017).

While there is evidence that exercise has positive effects on cortical activity during the performance of balance tasks, less is known on potential detrimental effects of physical (PF) and/or mental fatigue (MF). PF has been defined as exercise-induced declines in muscle force due repetitive single-joint (i.e., local fatigue) or multi-joint movement tasks (i.e., general fatigue) resulting in reduced sensory afferents from types Ia and II fibers of muscle spindles (Paillard, 2012). In contrast, MF impairs attentional control and working memory due to the performance after prolonged or strenuous cognitive task performed (e.g., attention network test, continuous performance test, and Stroop-task) of at least 30 min (Holtzer et al., 2011; Lew and Qu, 2014; Wascher et al., 2014; Van Cutsem et al., 2017a; Hachard et al., 2020; Verschueren et al., 2020). Additionally, there is preliminary evidence that PF and MF conditions modulate brain oscillatory activity in the theta, alpha, and beta frequency bands in frontoparietal areas (Bailey et al., 2008; Craig et al., 2012; Wascher et al., 2014; John et al., 2020). In this context, changes in frontal theta activity, presumably originating in the anterior cingulate cortex, have been observed in various cognitive and motor tasks requiring concentration, attention, working memory and performance monitoring (Smith et al., 1999; Slobounov et al., 2009, 2013; Shou et al., 2012; Baumeister et al., 2013; Sipp et al., 2013; Hülsdünker et al., 2015a,b; Gebel et al., 2020). Furthermore, changes in alpha-2 power in central and parietal brain areas involving the somatosensory cortex and sensory association areas seem to indicate task-specific sensory information processing (Leocani et al., 1997; Smith et al., 1999; Shou et al., 2012; Baumeister et al., 2013; Sipp et al., 2013; Gebel et al., 2020), while changes in beta-1 and beta-2 power in fronto-central areas have been associated with movement planning and execution (Leocani et al., 1997; Gwin et al., 2011; Shou et al., 2012; Sipp et al., 2013).

Several studies have shown that PF caused increased postural sway and sway velocity during the performance of balance tasks as well as altered spinal reflexes and increased co-contractions (Springer and Pincivero, 2009; Zech et al., 2012; Bryanton and Bilodeau, 2016; Ritzmann et al., 2016; Sadowska and Krzepota, 2016; Hamacher et al., 2018; Bedo et al., 2020). In a review article on the effects of PF on postural control, Paillard (2012) concluded that local and general PF deteriorate afferent sensory information processing and motor output during the performance of balance tasks which may partly be compensated by the allocation of additional cognitive resources.

Only few studies have examined the effects of MF on balance performance (Deschamps et al., 2013; Lew and Qu, 2014; Hachard et al., 2020; Morris and Christie, 2020; Verschueren et al., 2020). For instance, Deschamps et al. (2013) investigated the impact of a psychomotor vigilance test on static balance in healthy male students aged 22 years. Fatigue-related increases in static balance were found during bipedal stance on a foam surface. More recently, Hachard et al. (2020) evaluated the effects of a 90 min AX-continuous performance task on static balance in healthy young adults with a mean age of 21 years. Following the MF protocol, increased regularity of the center of pressure (CoP) indicated less automated processes of postural control with increased cognitive contributions during bipedal stance. The authors argued that in a state of MF, which led to impaired attentional processing, an increased activation of cognitive resources was necessary to control and monitor balance. Hence, these findings suggest that a fatigue related decline in balance performance might be compensated by the activation of additional cognitive resources (Hachard et al., 2020). In this context, Donker et al. (2007) reported that, in addition to CoP regularity, CoP variability is also well-suited to measure cognitive involvement in postural control. However, there is a paucity of data on the effects of PF and MF on neural correlates of balance performance. The available studies examined the effects of PF or MF on different measures of physical fitness (e.g., counter movement jumping height and gait velocity) and cognitive function (e.g., working memory performance) as well as their neural correlates.

Data on cortical activity provide further insight into the relationship between cortical activity and balance and how this relation is affected by fatigue. Therefore, the objective of this study was to examine the acute effects of MF and PF on measures of postural sway (i.e., CoP displacements [CoP_*d*_], velocity [CoP_v_], variability of CoP_d_ and CoP_v_) and cortical activity during the performance of a demanding balance task in healthy young adults. Based on the relevant literature, we expected increases in measures of postural sway following both fatigue protocols (Paillard, 2012; Zech et al., 2012; Sadowska and Krzepota, 2016; Grobe et al., 2017; Hachard et al., 2020). More specifically, due to the specifics of the fatigue protocols, we expected stronger declines in balance performance after PF. Additionally, as cognitive processing (e.g., allocation of attentional resources) is involved in postural control, especially when balance tasks are more challenging, we expected that MF affects balance performance too but to a lesser degree. Furthermore, we hypothesized that PF and MF result in spectral power increases across fronto-parietal areas in the theta, alpha-2, beta-1, and beta-2 frequency band (Bailey et al., 2008; Craig et al., 2012; Wascher et al., 2014; John et al., 2020). More precisely, we expected alpha-2 frequency band power increases to be larger over areas involved in sensory information processing (i.e., parietal) following PF compared to MF due to reduced sensory feedback as a result of PF.

## Materials and Methods

### Participants

With reference to the study of Sadowska and Krzepota (2016) and the reported large effect size (η^2^ = 0.23) of PF on CoP_v_, an *a priori* power analysis was performed in G × Power (Version 3.1.9.2, University of Kiel, Germany) using the *F* test family (ANOVA repeated measures, within between interaction). The analysis revealed that a total sample size of *N* = 12 would be sufficient to find significant large-sized pre-post effects of PF on CoP_v_ (effect size *f* = 0.5, α = 0.05, power = 0.80), with an actual power of 0.88 (critical *F*-value = 4.96). Accordingly, 15 healthy sport science students (6 females) aged 20–33 years were enrolled in this study. The participants’ self-reported physical activity level was assessed using the International Physical Activity Questionnaire-short (IPAQ-SF). Table 1 shows the characteristics of the participants. Individuals were excluded from study participation if they had any neurological diseases, medications that may influence cortical activity, or lower limbs musculoskeletal injury (e.g., ankle sprain) 6 months prior to the start of the study. Written informed consent was obtained from all participants. The study was approved by the local ethics committee of the University of Potsdam (application no 12/2019) and followed the latest version of the Declaration of Helsinki.

**TABLE 1:** Participants’ characteristics.

	Total (*N* = 15)	Male (*n* = 9)	Female (*n* = 6)
			
	*M* (*SD*)	*M* (*SD*)	*M* (*SD*)
Age (years)	28.8 (3.4)	29.7 (2.8)	27.5 (4.0)
Body height (cm)	173.3 (9.1)	178.5 (4.6)	165.4 (8.9)
Body mass (kg)	70.0 (9.8)	74.3 (8.4)	63.6 (8.9)

	**Low**	**Medium**	**High**

Physical activity level (IPAQ-SF)	0	4 (2f/2m)	11 (4f/7m)

*IPAQ-SF, International Physical Activity Questionnaire Short Form; f, female; m, male.*

### Experimental Procedure

A single group cross-over design was used to examine the effects of PF and MF on postural sway and cortical activity. For this purpose, participants were invited to the biomechanics laboratory for two experimental sessions to test the effects of PF and MF separately. The two sessions were scheduled 1 week apart at the same time of day to account for potential fatigue-related confounds such as muscle soreness, intraday performance variability, and learning effects. The order of the fatigue protocol was randomized. Every test session started with the measurements of anthropometrics followed by EEG preparations. The electrode cap was fitted to the participant’s head and the gel-electrodes were prepared. The experimental procedure continued with a standardized and short (3 min) familiarization period to introduce the multi-directional balance board which was used for balance testing. More information on the specifics of the balance task can be found in the section “Balance task.” After the familiarization period, baseline EEG recording was performed for 3 min during quiet bipedal stand with eyes opened on stable surface. Baseline tests were realized at the beginning of both experimental sessions. Thereafter, participants performed pre-tests in unfatigued condition with 5 60-s trials on the multi-directional balance board. Subsequently, the participants completed the respective fatigue protocol. Immediately after the fatigue protocol, post-tests were scheduled. Time between the termination of the fatigue protocol and the start of the post-tests was approximately 30 s. The post-tests followed the same procedure as described above, i.e., 5 consecutive 60-s trials of the balance task. During all trials, measures of postural sway and EEG were synchronously recorded.

### Physical Fatigue Protocol

To induce PF, a repeated sit-to-stand protocol was selected that resembled an everyday activity (Helbostad et al., 2007; Bryanton and Bilodeau, 2016). Due to the applied fatigue protocol together with the young age of the study participants, weighted vests with a load corresponding to 30% of the individuals’ body mass were used to induce PF. During the PF task, participants kept their arms crossed in front of their chest, the back was erect, and the knees were in a 90° at the starting position and fully extended during erect stance. Participants were asked to stand up and sit down at a self-selected pace until task failure. A metronome was not used during the performance of the PF protocol to prevent task failure due to imposed pacing and not PF. The fatigue protocol was completed if participants were unable to perform the sit-to-stand task anymore. Time until failure was recorded.

### Mental Fatigue Protocol

To induce MF, participants completed a software-based attention network test with a total protocol duration of 30 min (Fan et al., 2002). According to Van Cutsem et al. (2017a), MF protocols should at least last 30 min to make sure that participants are actually fatigued. The attention network test is a demanding attentional task, which combines cued visual reaction time (RT) tasks and a flanker task to assess three attentional network components (i.e., alerting, orienting, and executive control). The attention network test protocol used in this study consisted of 480 trials divided in five experimental blocks with 96 trials (4 cue conditions * 2 target locations * 2 target directions * 3 flanker conditions * 2 repetitions) each and were preceded by 24-trial training block. During the MF protocol, participants were in seated position in front of a screen and were asked to quickly and accurately determine the pointing direction of an arrow on the screen. The left arrow key on a keyboard should be pressed with the left index finger for leftward pointing arrows and the right arrow key with the right index finger for rightward pointing arrows. The arrow could appear above or below a fixation cross and might or might not be accompanied by different cue conditions or flanker stimuli. Flankers consisted of neutral dashes (e.g., --→--) or a sequence of congruent (e.g., →→→→→) or incongruent arrows (e.g., ←←→←←). The four cue conditions were (i) no cue, (ii) center cue, (iii) double cue (no directed spatial information) and (iv) spatial cue (Fan et al., 2002). Subtractions of performance (error rate, RTs) between the different stimulus conditions reflect specific attentional networks and were performed according to Holtzer et al. (2011): no cue – center cue for alerting, center cue – spatial cue for orienting and incongruent vs. congruent flanker trials for executive attention. The mean error rates and reaction times across all blocks were taken to assess performance during the MF protocol. Additionally, mean attention network scores (i.e., alerting, orienting, and executive) were calculated for each of the five blocks and analyzed to identify a potential fatiguing effect on specific attentional networks (Holtzer et al., 2011).

### Subjective Level of Physical and Mental Fatigue

In order to evaluate the subjective levels of PF/MF, participants were asked to rate their perceived levels of fatigue on a visual analogue scale (VAS) from 0 cm (not physically/mentally fatigued at all) to 10 cm (extremely physically/mentally fatigued) as previously reported by Van Cutsem et al. (2017b) and Verschueren et al. (2020). Subjective levels of fatigue were assessed immediately before and after the respective fatigue protocol.

### Balance Task

The balance tests required participants to stand as still as possible in bipedal and barefooted stance for 60 s on a commercially available multi-directional balance board (Wobblesmart^©^, Artzt GmbH, Dornburg, Germany). Test trials were started with the balance board in quiet and horizontal position. While performing the balance task, participants were instructed to place their hands akimbo and to fix a cross on a nearby wall (3 m distance) with their eyes. Participants were kindly instructed to avoid tilting movements as well as ground contacts of the board. In other words, participants’ task was to keep the board in horizontal position (Gebel et al., 2019). In brief, the pivot of the balance board has 6 difficulty levels. While level 1 is characterized by the largest base of support, level 6 has the smallest. During all tests, level 4 was selected. Two sensor mats (Pedar^©^, novel GmbH, Posturo S2094, novel GmbH, Munich, Germany) were placed on top of the balance board to measure CoP_d_ and CoP_v_ at a sampling frequency of 40 Hz. Postural data were analyzed using the manufacturer software (Posturo 32 Expert software, version 25.3.6, novel GmbH, Munich, Germany). Biomechanical and neurophysiological data were synchronized by sending a continuous 5 V signal from the Pedar^©^ system (Posturo Sync Box, novel GmbH, München, Germany) to the EEG system from the start to the end of each test trial. For additional analyses of CoP variability, the respective coefficients of variation (CV) were calculated for CoP_d_ and CoP_v_.

### Electroencephalography Recordings and Analysis

Cortical activity was continuously recorded during each balance test trial. Therefore, a mobile EEG system (eego™ sports, Advanced Neuro Technology B.V., Enschede, Netherlands) with 64 Ag/AgCl passive gel-electrodes implemented in an elastic cap (Waveguard classic, Advanced Neuro Technology B.V., Enschede, Netherlands) was used. Electrodes inside the cap were positioned in conformance with the extended 10–20 system. All channels were re-referenced to the CPz electrode. To obtain a high signal-to-noise ratio, electrode impedances were kept below 5 kΩ. EEG signals were amplified, digitized with a 24-bit analog-to-digital converter (eego™, Advanced Neuro Technology B.V., Enschede, Netherlands) and recorded with the eego™ software (Version 1.2.1, Advanced Neuro Technology B.V. Enschede, Netherlands) at a sampling frequency at 1,024 Hz. Offline EEG data processing was performed using the EEGLAB 13.5.4b toolbox (Delorme and Makeig, 2004) implemented in MATLAB (MathWorks Inc., Natick, MA, United States). Sinusoidal line noise was removed by means of the CleanLine plugin (Mullen, 2012). EEG signals were band pass filtered with a finite impulse response filter ranging from 3 to 50 Hz and finally down-sampled to 256 Hz. Channels contaminated by high-frequency noise, electrode movement, and non-stereotypical electromyographic activity were manually removed. EEG data were then re-referenced to common average. Continuous data were visually inspected, and the identified non-stereotypical artifacts were removed from the data set. An adaptive mixture independent component analysis (Palmer et al., 2011) was performed on the remaining data to identify and remove independent components representing stereotypical artifacts like electro-oculographic (i.e., eye blinks) sources, muscle electromyographic activities (Onton and Makeig, 2006). For frequency specific analyses, EEG data were merged for all five trials recorded before (pre) and after (post) the respective fatigue protocol. This was done for each fatigue protocol and participant separately. According to previous studies (Slobounov et al., 2008, 2013; Hülsdünker et al., 2015a,b; Edwards et al., 2018; Gebel et al., 2020), three regions of interest (ROI) were built at frontal (F3, F1, Fz, F2, and F4), central (C3, C1, Cz, C2, and C4), and parietal (P3, P1, Pz, P2, and P4) areas of the cortex associated with processing attention (Shou et al., 2012; Baumeister et al., 2013; Hülsdünker et al., 2015a,b; Gebel et al., 2020), motor planning and execution (Leocani et al., 1997; Gwin et al., 2011; Shou et al., 2012; Sipp et al., 2013), and sensory information (Leocani et al., 1997; Shou et al., 2012; Baumeister et al., 2013; Gebel et al., 2020). Absolute spectral power was calculated for theta (4–7.5 Hz), alpha-2 (10.5–12.5 Hz), beta-1 (13–18 Hz), and beta-2 (18.5–25 Hz) in the respective ROIs using a fast Fourier transformation with a spectral resolution of 1 Hz and a 10% Hanning window. For further analyses, spectral power values were then normalized to a baseline recording taken prior to the tests during unfatigued bipedal standing on stable surface.

### Statistical Analyses

Data are presented as mean and standard deviation. Distribution of the data for normality was checked by the Shapiro–Wilk test. To analyze fatigue-related pre-post differences in measures of balance and fatigue, five separate two-way repeated measures analysis of variance (rmANOVA) were performed for CoP_v_, total CoP_d_, CV CoP_v_, CV CoP_d_ and VAS scores. Moreover, three rmANOVAs were calculated to evaluate attention network test performance (i.e., error rates, reaction times, and attention network scores) across all participants and blocks. Finally, twelve two-way rmANOVAs were calculated to examine the potential effects of fatigue on frequency-specific cortical activity in the four frequency bands within the three predefined ROIs (frontal, central, and parietal). If significant time-by-fatigue interactions were found, *post hoc* Holm–Bonferroni adjusted paired *t*-tests were computed. In addition, Pearson correlation coefficients were calculated between measures of postural sway and spectral power for frequencies with significant time-by-fatigue interactions. All effect sizes are presented as Cohens *d*. If necessary, effect estimates (η^2^) were converted accordingly. As proposed by Cohen (1988), an effect was considered small with an effect size of *d* ≥ 0.2, medium *d* ≥ 0.5 and large *d* ≥ 0.8. The statistical analyses were calculated using the JASP statistical software (Version 0.14.1.0; JASP Team, 2020).

## Results

### Subjective Level of Physical/Mental Fatigue

**FIGURE 1:**
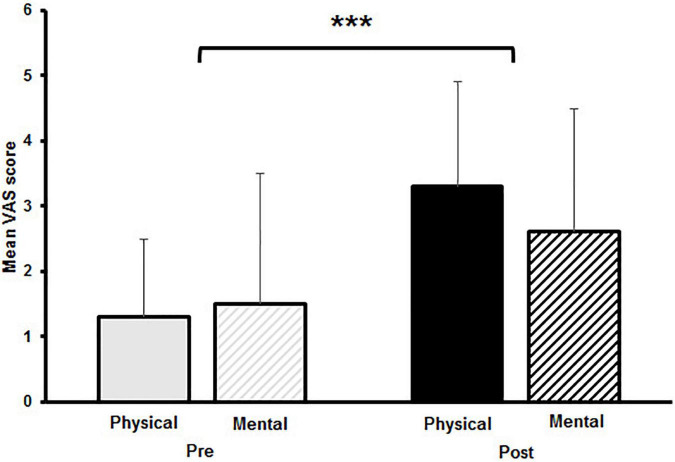
Mean visual analogue scale (VAS) scores with standard deviation of subjective levels of fatigue pre and post to physical and mental fatigue. The significant main effect for time is indicated by the bracket with asterisk; ***p < 0.001.

Averaged VAS scores are shown in Figure 1. A significant main effect for time was observed for the VAS scores [*F*_(1,14)_ = 31.431, *p* < 0.001, *d* = 0.91]. No main effect for fatigue [*F*_(1,14)_ = 0.943, *p* = 0.35] and no interaction effect for time and fatigue [*F*_(1,14)_ = 1.934, *p* = 0.186] was found, indicating comparable changes in subjective fatigue after the PF and the MF protocol.

### Physical and Mental Fatigue Protocol

**FIGURE 2:**
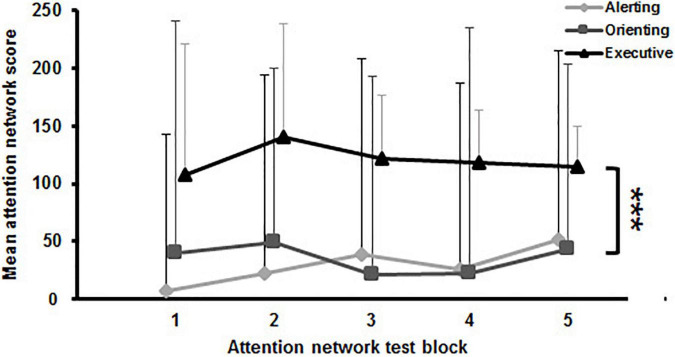
Mean attention network scores with standard deviation of the attention network test across blocks. The significant main effect for network is indicated by the bracket with asterisk; ***p < 0.001.

During the PF protocol, the time until exhaustion was clocked for each participant individually. The average time to exhaustion was 10 min (±5.5) with a range from 2 to 21 min. Regarding the MF protocol, the average error rates [*F*_(2.2,10.1)_ = 1.069, *p* = 0.361] and reaction times [*F*_(3.9,5608.7_) = 1.805, *p* = 0.126] across all participants between blocks showed no significant differences (Table 2). Analyses of the mean attention network scores (i.e., alerting, orienting, and executive) across blocks revealed significant main effect for network [*F*_(2,28)_ = 18.053, *p* < 0.001, *d* = 2.27]. Scores for the executive attention network were significantly higher than for the alerting [*t*_(74)_ = 6.59, *p* < 0.001, *d* = 1.70] and orienting network [*t*_(74)_ = 4.01, *p* = 0.003, *d* = 1.04] (Figure 2). However, no main effect for block [*F*_(1.4,8828.7)_ = 1.010, *p* = 0.356] and no interaction effect for block and network [*F*_(2.7,8345.1)_ = 0.957, *p* = 0.416] was found. Thus, while an increase in subjective fatigue was reported in the VAS, behavioral performance in the attention network test did not deteriorate significantly over time.

**TABLE 2:** Error rates and reaction times for the different blocks of the attention network test presented as mean with standard deviation across all participants.

	Block 1	Block 2	Block 3	Block 4	Block 5
					
	*M* (*SD*)	*M* (*SD*)	*M* (*SD*)	*M* (*SD*)	*M* (*SD*)
Error rates (%)	3.7 (3.4)	2.4 (2.1)	2.8 (1.6)	2.9 (1.8)	2.1 (1.9)
Reaction time (ms)	582.5 (166.9)	577.5 (158.0)	577.5 (161.0)	570.5 (178.9)	569.7 (161.4)

### Balance Performance

**FIGURE 3:**
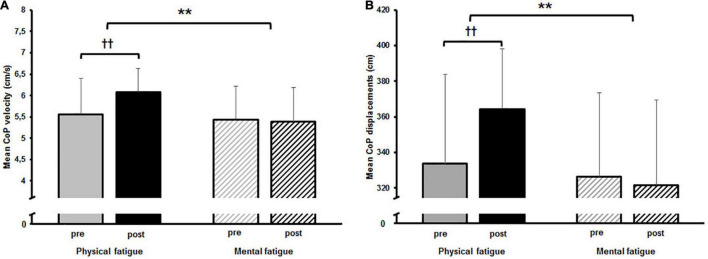
**(A)** Mean center of pressure (CoP) velocity and **(B)** mean CoP displacement pre and post to both fatigue protocols as well as the results of the *post hoc* comparisons. The significant interaction effect is indicated by; **p < 0.01. *Post hoc* results are indicated by; ^†⁣†^*p* < 0.01.

**FIGURE 4:**
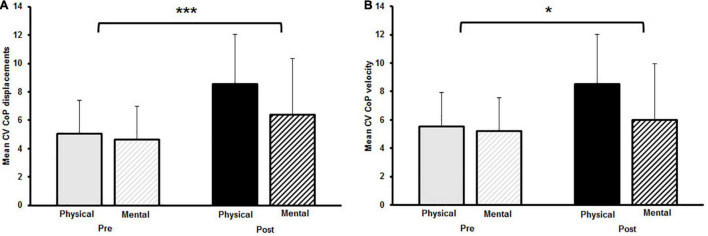
**(A)** Mean coefficient of variation (CV) for center of pressure (CoP) displacement and **(B)** mean CV CoP velocity pre and post to physical and mental fatigue. The significant main effect for time is indicated by the bracket with asterisk; *p < 0.05, ***p < 0.001.

The two-way rmANOVA revealed a significant main effect for time on CoP_v_ [*F*_(1,14)_ = 6.789, *p* = 0.021, *d* = 0.3]. Moreover, a significant small-sized time-by-fatigue interaction was indicated [*F*_(1,14)_ = 9.062, *p* = 0.009, *d* = 0.36]. *Post hoc* tests showed a significant increase of sway velocity following PF [*t*_(29)_ = 3.98, *p* = 0.003, *d* = 1.03, Δ 9.2%] while sway velocity remained on pre-fatigue level after MF [*t*_(29)_ = 0.4, *p* = 1.00, Δ−1.0%] (Figure 3A). The statistical analyses for CoP_d_ yielded a significant main effect for fatigue [*F*_(1,14)_ = 8.677, *p* = 0.011, *d* = 0.55] and time [*F*_(1,14)_ = 6.683, *p* = 0.022, *d* = 0.28]. Additionally, the analyses revealed a small-sized time-by-fatigue interaction [*F*_(1,14)_ = 9.197, *p* = 0.009, *d* = 0.39] for postural sway. *Post hoc* analyses showed a significant increase of postural sway following the PF [*t*_(29)_ = 3.98, *p* = 0.002, *d* = 1.03, Δ 9.2%] but not the MF protocol [*t*_(29)_ = 0.62, *p* = 0.97, Δ−1.5%] (Figure 3B). In terms of CoP variability, the analyses revealed a significant large-sized main effect of time for CV CoP_d_ [*F*_(1,14)_ = 16.342, *p* = 0.001, *d* = 0.84] (Figure 4A) as well as a significant medium-sized main effect of time for CV CoP_v_ [*F*_(1,14)_ = 4.617, *p* = 0.05, *d* = 0.62] (Figure 4B). No main effects of fatigue or interactions were found.

### Cortical Activity

**FIGURE 5:**
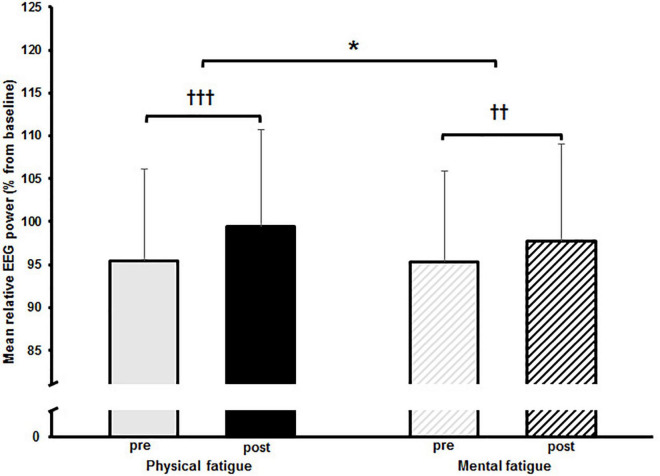
Mean relative alpha-2 frequency band power with standard deviation pre and post to physical and mental fatigue calculated from the electroencephalographic (EEG) electrodes located within the parietal region of interest. The significant interaction effect is indicated by; *p < 0.05. *Post hoc* results are indicated by; ^†⁣†^*p* < 0.01, ^†⁣†⁣†^*p* < 0.001.

The two-way rmANOVAs with factors time and fatigue indicated a significant main effect of time for relative theta power in the central [*F*_(1,14)_ = 8.648, *p* = 0.011, *d* = 0.09] and parietal ROI [*F*_(1,14)_ = 6.646, *p* = 0.022, *d* = 0.09], for relative alpha-2 power in the frontal [*F*_(1,14)_ = 27.812, *p* < 0.001, *d* = 0.19], central [*F*_(1,14)_ = 28.144, *p* < 0.001, *d* = 0.14] and parietal ROI [*F*_(1,14)_ = 35.879, *p* < 0.001, *d* = 0.26], for relative beta-1 power in the frontal [*F*_(1,14)_ = 14.005, *p* = 0.002, *d* = 0.09], central [*F*_(1,14)_ = 20.543, *p* < 0.001, *d* = 0.11] and parietal ROI [*F*_(1,14)_ = 17.785, *p* < 0.001, *d* = 0.13], and for relative beta-2 power in the parietal ROI [*F*_(1,14)_ = 5.207, *p* = 0.039, *d* = 0.11] (Tables 3–6). Moreover, a significant time-by-fatigue interaction [*F*_(1,14)_ = 7.149, *p* = 0.018, *d* = 0.06] was found for the parietal ROI. Applied *post hoc t*-tests indicated significant increases in relative alpha-2 power (Figure 5) during balance task performance after both fatigue protocols [physical: *t*_(29)_ = 6.54, *p* < 0.001, *d* = 1.69; mental: *t*_(29)_ = 3.98, *p* = 0.003, *d* = 1.03]. The relative power increase was more pronounced after the PF (Δ 3.9%) than the MF protocol (Δ 2.5%). Further, we checked whether the interaction effect was confounded by a potential retest effect. The dependent t-test between pre-test values of parietal alpha-2 indicated no significant difference [*t*_(29)_ = −0.174, *p* = 0.864]. Calculation of the intraclass correlation coefficient (ICC; 2-way mixed model, single measurement) according to Koo and Li (2016) also yielded good test-retest reliability with an ICC of 0.82, 95% confidence interval (CI) [0.54, 0.94] for relative alpha-2 frequency band power. Additionally, the analyses yielded a tendency toward significance for a time-by-fatigue interaction [*F*_(1,14)_ = 4.260, *p* = 0.058] within the central ROI for relative alpha-2 frequency power. No main effects for fatigue or other interactions were found (Tables 3–6).

**TABLE 3:** Averaged theta frequency power relative to baseline values pre and post to PF and MF within the respective ROIs as well as rmANOVA results and *post hoc* tests.

	Relative theta power			RmANOVA			*Post hoc*	
				
	Pre	Post	Change (%)	Time	Fatigue	Time*fatigue	*p*-value	Effect size (*d*)
	
	*M* (*SD*)	*M* (*SD*)						
**Frontal**								

Physical	101.5 (13.2)	101.5 (12.8)	0.0	*p* = 0.228	*p* = 0.789	*p* = 0.184	–	–
Mental	100.7 (12.7)	101.8 (13.9)	1.1				–	–

**Central**								

Physical	101.0 (11.2)	101.8 (11.0)	0.8	***p* = 0.011**	*p* = 0.782	*p* = 0.579	–	–
Mental	101.4 (14.0)	102.6 (14.6)	1.2				–	–

**Parietal**								

Physical	100.4 (11.8)	101.6 (11.9)	1.2	***p* = 0.022**	*p* = 0.656	*p* = 0.685	–	–
Mental	99.9 (11.2)	100.9 (12.6)	1.0				–	–

*Data are presented as M with SD in % from baseline. Bold p-values indicate statistical significancy.*

*Post hoc tests were computed if the omnibus test (i.e., time-by-fatigue interaction) turned out to be significant.*

**TABLE 4:** Averaged alpha-2 frequency power relative to baseline values pre and post to PF and MF within the respective ROIs as well as rmANOVA results and *post hoc* tests.

	Relative alpha-2 power				RmANOVA			*Post hoc*	
				
	Pre	Post	Change (%)	Time	Fatigue	Time*fatigue	*p*-value	Effect size (*d*)
	
	*M* (*SD*)	*M* (*SD*)						
**Frontal**								

Physical	97.2 (17.1)	100.2 (13.0)	3.0	***p* < 0.001**	*p* = 0.475	*p* = 0.379	–	–
Mental	96.1 (15.6)	98.5 (16.7)	2.4				–	–

**Central**								

Physical	96.6 (13.5)	99.6 (13.9)	3.0	***p* < 0.001**	*p* = 0.746	*p* = 0.058	**–**	–
Mental	96.0 (18.7)	100.8 (18.8)	4.8				–	–

**Parietal**								

Physical	95.5 (10.6)	99.4 (11.3)	3.9	***p* < 0.001**	*p* = 0.512	***p* = 0.018**	**<0.001**	1.69
Mental	95.3 (12.8)	97.7 (14.1)	2.5				**0.003**	1.03

*Data are presented as M with SD in % from baseline. Bold p-values indicate statistical significancy.*

*Post hoc tests were computed if the omnibus test (i.e., time-by-fatigue interaction) turned out to be significant.*

**TABLE 5:** Averaged beta-1 frequency power relative to baseline values pre and post to PF and MF within the respective ROIs as well as rmANOVA results and *post hoc* tests.

	Relative beta-1 power				RmANOVA			*Post hoc*	
				
	Pre	Post	Change (%)	Time	Fatigue	Time*fatigue	*p*-value	Effect size (*d*)
	
	*M* (*SD*)	*M* (*SD*)						
**Frontal**								

Physical	101.0 (12.8)	101.9 (12.6)	0.9	***p* = 0.002**	*p* = 0.810	*p* = 0.591	–	–
Mental	100.3 (14.3)	101.8 (15.8)	1.5				–	–

**Central**								

Physical	99.9 (12.1)	101.5 (12.1)	1.6	***p* < 0.001**	*p* = 0.807	*p* = 0.881	–	–
Mental	100.6 (17.4)	102.1 (17.4)	1.5				**–**	–

**Parietal**								

Physical	99.2 (11.4)	101.2 (11.1)	2.0	***p* < 0.001**	*p* = 0.824	*p* = 0.246	–	–
Mental	99.4 (12.0)	100.4 (13.5)	1.0				–	–

*Data are presented as M with SD in % from baseline. Bold p-values indicate statistical significancy.*

*Post hoc tests were computed if the omnibus test (i.e., time-by-fatigue interaction) turned out to be significant.*

**TABLE 6:** Averaged beta-2 frequency power relative to baseline values pre and post to PF and MF within the respective ROIs as well as rmANOVA results and *post hoc* tests.

	Relative beta-2 power			RmANOVA			*Post hoc*	
				
	Pre	Post	Change (%)	Time	Fatigue	Time*fatigue	*p*-value	Effect size (*d*)
	
	*M* (*SD*)	*M* (*SD*)						
**Frontal**								

Physical	102.0 (12.6)	101.3 (12.1)	−0.7	*p* = 0.437	*p* = 0.703	*p* = 0.194	–	–
Mental	100.6 (13.0)	101.8 (13.8)	1.2				–	–

**Central**								

Physical	99.2 (9.9)	99.9 (9.8)	0.7	*p* = 0.09	*p* = 0.743	*p* = 0.653	–	–
Mental	99.8 (16.2)	100.9 (14.3)	1.1				**–**	–

**Parietal**								

Physical	100.0 (9.4)	101.2 (8.6)	1.2	***p* = 0.039**	*p* = 0.601	*p* = 0.843	–	–
Mental	99.5 (9.4)	100.4 (10.6)	0.9				–	–

*Data are presented as M with SD in % from baseline. Bold p-values indicate statistical significancy.*

*Post hoc tests were computed if the omnibus test (i.e., time-by-fatigue interaction) turned out to be significant.*

Finally, Pearson correlation coefficients were calculated between pre/post changes (deltas) for measures of postural sway (i.e., CoP_d_, CoP_v_, CV CoP_d_, and CV CoP_v_) and relative alpha-2 power of the parietal ROI. No statistically significant associations were found between balance performance and cortical activity, irrespective of the fatigue protocol. The respective correlation coefficients for PF between alpha-2 power and measures of postural sway were *r*_(13)_ = 0.191, (*p* = 0.496, CI −0.37, 0.64) for CoP_d_ and CoP_v_, *r*_(13)_ = 0.004 (*p* = 0.989, CI −0.51, 0.51) for CV CoP_d_, and *r*_(13)_ = −0.152 (*p* = 0.588, CI −0.62, 0.39) for CV CoP_v_. Regarding MF, the correlation analyses for alpha-2 power with both measures of postural sway resulted in *r*_(13)_ = −0.267 (*p* = 0.335, CI −0.69, 0.28) for CoP_d_, *r*_(13)_ = −0.351 (*p* = 0.200, CI −0.73, 0.20) for CoP_v_, *r*_(13)_ = −0.134 (*p* = 0.633, CI −0.60, 0.41) for CV CoP_d_, and *r*_(13)_ = −0.403 (*p* = 0.136, CI −0.76, 0.14) for CV CoP_v_.

## Discussion

This study is the first to compare the effects of a PF and MF on cortical activity in the theta, alpha, and beta frequency bands as well as on postural sway while performing a challenging balance task in healthy young adults. The main findings of this study were that only the PF protocol affected postural sway (CoP displacements) and sway velocity while sway variability and cortical activity were affected by both, mental and physical fatigue. In terms of postural sway and sway velocity, CoP_d_ and CoP_v_ increased significantly only after the physical fatigue protocol using repeated sit-to-stand tasks to failure. Both fatigue protocols had an impact on variability of postural sway and sway velocity (i.e., CV CoP_d_ and CV CoP_v_). No statistically significant time-by-fatigue interaction was observed for relative theta, beta-1, and beta-2 frequency band power in the three ROIs (i.e., frontal, central, and parietal). However, the relative alpha-2 frequency band power increased significantly in the parietal ROI after both fatigue protocols with a significantly larger increase after the PF protocol. This could reflect, amongst others, deteriorated sensory information processing related to impaired balance performance caused by PF.

### Effects of Fatigue on Balance Performance

The participants reported to have reached a state of fatigue after both, the PF and MF protocol, as indicated by the significant pre/post increase in the VAS scores. Additionally, analyses of the attentional network scores of the attention network test indicated a higher attentional load within the executive attention network without an observable fatiguing effect. Even though participants stated to be physically and mentally fatigued, the observed effects on postural sway diverged from these results. In accordance with our first hypothesis, we observed increased postural sway (CoP_d_), sway velocity (CoP_v_), and sway variability (CV CoP_d_ and CV CoP_v_) during quite bipedal standing on a wobble board after performing a modified sit-to-stand task till exhaustion in healthy young adults. These results indicate the negative effects of PF on balance performance, although Santos et al. (2019) point out that fatigue-related changes after multi joint exercise are the result of combined physiological and mental fatiguing effects.

Nonetheless, our results are in line with previous research, which reported fatigue-related impairments for balance performance and, thus, postural control resulting in significant increases in postural sway (Sadowska and Krzepota, 2016; Penedo et al., 2021), sway velocity (Zech et al., 2012), sway area (Bedo et al., 2020; Moon et al., 2020; Penedo et al., 2021), as well as decreases of the stability index (Cooper et al., 2020) during stable and unstable bipedal and unipedal stance in healthy young adults. For instance, Zech et al. (2012) investigated the effects of a localized fatigue versus a general fatigue protocol on static and dynamic balance in male handball athletes. The authors reported significant increases in sway velocity during single leg stance after both fatigue protocols while no changes were observed in star excursion balance test performance. Accordingly, they assumed that there might exist different sensorimotor control mechanisms within the postural control system responsible for static and dynamic balance which are affected differently by the applied fatigue protocols. Moreover, Penedo et al. (2021) examined the effects of local PF on postural sway and lower limb muscle activation in healthy young adults. While postural sway increased, no changes in muscle activation were observed after PF. The authors suggested that impairments in postural control emerge from deteriorated proprioception as well as from changes within the peripheral and central systems (Penedo et al., 2021).

In this context, Gandevia (2001) and Paillard (2012) described in their extensive literature reviews potential spinal (e.g., altered sensory input) and supraspinal factors (e.g., changes in cortical excitability and inhibitory processes) contributing to the fatigue-related declines in afferent sensory information processing and motor output of postural control. The potential role of these factors is discussed together with the changes in cortical activity related to balance performance decreases.

With respect to the effects of MF on postural control, we did not observe significant increases in postural sway (CoP_d_) and sway velocity (CoP_v_). At first glance, these results seem to be contradicting previously reported findings. Morris and Christie (2020), for instance, found increased sway velocity during a bipedal reactive balance task in young women after performing a psychomotor vigilance task for 20 min. Similarly, Deschamps et al. (2013) showed significantly increased body sway during bipedal stance on a foam surface after inducing MF. However, and despite the absence of a decrease in balance performance, we observed increases in sway variability (CV CoP_d_ and CV CoP_v_) after the MF protocol suggesting that postural control was influenced by mental fatigue. These results are consistent with findings by Hachard et al. (2020) who investigated the effect of MF on static balance in healthy young adults. After performing a 90 min continuous performance task, the authors found no changes in sway velocity and sway area whereas dynamical features of postural sway variations (i.e., CoP sample entropy) decreased during quiet bipedal stance. Decreases in sample entropy were interpreted as increased cognitive contributions to postural control to compensate for the impact of fatigue and to maintain balance performance. Moreover, Noé et al. (2021) reported that the completion of the same prolonged demanding cognitive task (i.e., continuous performance task) induces a strong heterogeneity in subjects’ responses, which affect the individual’s postural control system differently according to the sensory context. Thus, it seems that the effects of MF on postural control are initially not reflected by crude CoP measures such as displacement or velocity but by CoP variability or non-linear measures like sample entropy. In addition, the individual’s responses to the MF protocol and consequently its different effects on postural control might have confounded our results regarding postural sway and sway velocity. The short duration of the attention network test protocol might also have a confounding effect, but this is discussed in the limitation section.

Although we did not observe a clear deterioration in balance performance after a prolonged cognitive task, the increase in CoP variability can be interpreted as increasing cognitive involvement in postural control (Donker et al., 2007). Moreover, our results consolidate previous findings on the fatigue-inducing effects of multi-joint exercises resulting in impairments of postural control. Future studies should include analyses of CoP parameters such as CoP variability or CoP regularity (Hachard et al., 2020) as traditional CoP parameters (e.g., CoP_d_) tend to be less sensitive to detect initial changes in postural control due to MF.

### Effects of Fatigue on Cortical Activity

In terms of cortical activity, our results reveal widespread increases in relative alpha-2 and beta-1 frequency band power in combination with more restricted increases in relative theta (i.e., central and parietal) and beta-2 power (i.e., parietal) after both fatigue protocols. Previous studies examining the effects of PF on cortical activity reported increases in theta, alpha, and beta frequency band power at frontal, central, and parietal brain areas (Crabbe and Dishman, 2004; Bailey et al., 2008; Dishman et al., 2010; John et al., 2020). Moreover, MF studies reported increases in fronto-central theta power (Craig et al., 2012; Tanaka et al., 2012; Wascher et al., 2014; Fan et al., 2015) as well as increases in the alpha (Craig et al., 2012; Fan et al., 2015) and beta power (Craig et al., 2012) in fronto-central and parieto-occipital areas after mental fatiguing tasks in healthy adults.

For example, Bailey et al. (2008) investigated the influence of a graded exercise test until exhaustion on cortical activity at frontal, central, and parietal electrode sites. Their results indicated increases in theta, alpha-1, alpha-2, beta-1, and beta-2 frequency band power relative to baseline across selected frontal, central, and parietal areas. Similar findings were obtained by John et al. (2020), who also observed increases in theta, alpha, and beta frequency band power at frontal, central, and parietal brain areas during and after performing a graded exercise test until exhaustion. The authors suggested that stronger cortical involvement might reflect adaptive mechanisms to maintain information processing and to cope with fatigue-induced impairments by increasing attentional processes.

Regarding MF, Wascher et al. (2014) examined the effects of a prolonged spatial stimulus-response-compatibility task on cortical activity in healthy young adults. Results showed a continuous increase of theta power at electrodes over fronto-central areas of the cortex as well as an increase of alpha-1 and alpha-2 power at fronto-central electrode sites with longer duration of time on task. Moreover, Craig et al. (2012) examined changes in EEG measures that occurred during a driving simulator task in healthy adults. With proceeding MF, spectral power within the theta, alpha-1, alpha-2, beta-1, and beta-2 frequency band increased in frontal, central, and parietal areas and were accompanied by decreasing task performance. Thus, Craig et al. (2012) assumed that a fatigue-induced decline in cognitive capacity associated with impaired task performance might be related to global increase in theta, alpha 1, and alpha 2 wave power.

However, when comparing the present results with previous findings, it should be borne in mind that previous studies investigated fatigue-induced changes on cortical activity in sedentary participants at rest and not during performance of a challenging balance task. Widespread increases in relative alpha-2 and beta-1 frequency band power in combination with the more localized increases in relative theta (i.e., central and parietal) and beta-2 power (i.e., parietal), therefore, might reflect fatigue-related changes in cortical network activity involved in postural control (Solis-Escalante et al., 2019; Varghese et al., 2019). More specifically, changes in alpha-2 power over centro-parietal areas could be linked to balance performance decreases after PF. However, it should be noted that pre-post changes (deltas) in CoP variables and alpha-2 power did not correlate significantly with each other. Accordingly, the interpretation of the cortical mechanisms behind the observed fatigue-related effects on balance performance remain speculative and further research is needed to elucidate this issue. According to the literature, declines of alpha-2 power in centro-parietal areas, where the sensorimotor cortex and precuneus are localized, represent task specific sensory and movement-related information processing (Leocani et al., 1997; Neuper and Pfurtscheller, 2001). Consequently, the observed pre to post increases of relative alpha-2 power in these areas with concomitant decreases in balance performance might indicate deterioration of those processes. This deterioration might result in impaired sensory information processing related to movement planning and execution during balance control (Robertson and Marino, 2015).

Another explanation for the large increases of parietal alpha-2 power might be increased activity of somatosensory afferents after exercise (Dishman et al., 2010). Altered sensory input from muscle spindles and tendon organs (Paillard, 2012) as well as from muscle afferents (i.e., groups III and IV) that innervate the fatigued muscles (Gandevia, 2001) during the postural task, thus, might explain changed activity at central and parietal electrode sites overlaying portions of the somatosensory cortex and the precuneus, a structure known to be involved in dynamic balance control revealed by imaging data (Papegaaij et al., 2017).

Moreover, Benedek et al. (2014) suggested that parietal alpha power increases might reflect an inhibition of the ventral attention network which is thought to prevent reorienting to irrelevant stimulation during goal-driven, top-down behavior. This in turn might be indicative for a shielding function during cognitive demanding tasks to avoid/minimize distractor interference.

Thus, parietal alpha-2 power increases following the MF and PF protocol might represent shielding of specific cortical areas in an effort to maintain relevant task-specific information processing. It might also be possible that all, a shielding function, increased sensory afferent input and impaired task specific sensory, and movement-related information processing, are held responsible for the observed increments in alpha-2 power. As brain dynamics are inherently multi-scale, the underlying cortical mechanisms for the observed findings on balance decrements with fatigue cannot be elucidated with this study. Therefore, future studies should further investigate the role of centro-parietal alpha-2 activity in fatigue condition and if it is linked to changes in balance performance utilizing high-density EEG and source localization. Moreover, these studies should address other fatigue-related spectral EEG measures such as individual alpha peak frequency (Ng and Raveendran, 2007; Mierau et al., 2017). In this regard, also lateralization effects are of particular interest together with the specific effects of physical versus mental fatigue protocols on spectral EEG measures (e.g., alpha peak frequency).

### Limitations

First, PF was assessed by means of a VAS reflecting only the subjective feeling of PF. The additional assessment of heart rate or blood lactate would have provided an objective measure to determine whether participants reached exhaustion. However, the PF protocol can be rated as effective as observed performance impairments were in line with the literature. Further, one could argue that the range from 2 to 21 min until task failure is large and might have confounded our results. As almost all participants had a high level of physical activity and participants were selected from a rather homogeneous cohort of sport science students, we consider that strength and fitness did not have an impact on the outcomes of this study.

Second, we had no control condition in which participants had to rest for a certain time (e.g., 30 min). Therefore, even though we controlled for a re-test effect, we cannot completely rule out that increases in relative frequency band power during performance of challenging balance task are solely attributable to the fatigue protocols. As previous controlled studies reported similar increases, it can be argued that the observed changes in cortical activity are very likely a result of PF and MF. Moreover, the time (∼30 s) between the termination of the fatigue protocol and the starting of post-tests could have been too long so that maybe fatigue-related effects were mitigated. We also did not have a seated control test measurement of EEG activity hence we cannot tell if the changes we report here are specific to the balance task or could have been present in sitting as well. There was little specificity between the fatigue tasks and the balance board test task. We cannot tell if fatigue would have been induced by a balance task and tested with the same balance task, the effects of PF would have been more profound on EEG spectral power.

Third, one can argue that the duration of the attention network test might have been too short to induce MF. In this context, Holtzer et al. (2011) were able to show that even a 30-min attention network test has a fatiguing effect on the executive attention network. Since the authors examined old adults in their study, it seems possible that the test duration would have to be slightly longer to induce MF in young adults. However, even though balance performance (i.e., CoP_d_ and CoP_v_) was not impaired, CoP variability increased after both fatigue protocols. The observed changes in cortical activity, especially increases in alpha-2 power, therefore, seem to be indicative for a compensatory mechanism (Benedek et al., 2014) to maintain relevant task-specific information processing and thus performance during fatigue. Similarly, Hachard et al. (2020) observed increased cognitive contributions (i.e., decreased sample entropy) to postural control while balance performance remained unchanged. Consequently, balance performance must not be necessarily impaired in a state of MF if sufficient cognitive resources can be allocated to compensate for the negative impact of fatigue on postural control.

Finally, it has to be noted that causality between changes in alpha-2 power over centro-parietal areas with measures of balance after PF remains speculative due to the absence of a significant correlation between changes in alpha-2 power and CoP variables. However, Hedge et al. (2018) argued that the reliability of the single behavioral and neurophysiological measures might be a problem with such correlative analyses. Furthermore, inferences from electrode signals to the origin of changes in the oscillatory activity of the brain are speculative, as the brain works as a volume conductor. Therefore, more studies utilizing high-density EEG, source localization, and co-registration are needed in the future to investigate a possible relation between the effects of PF and MF on balance performance and cortical activity.

## Conclusion

In summary, the present study revealed decreased balance performance indicated by increased CoP_d_ and CoP_v_ after performing a PF but not MF protocol. In addition, MF and PF resulted in increased CoP variability. Cortical activity in the form of relative frequency band power increased across almost all ROIs and frequency bands, irrespective of the fatigue protocol under investigation. Notably, increases in relative alpha-2 power in the parietal ROI were significantly larger after PF. These increases in parietal alpha-2 power could reflect a fatigue-induced impairment of sensory information processing related to movement planning and execution within the somatosensory cortex and precuneus resulting in decreased balance performance and impaired postural control. However, other underlying cortical mechanisms including a shielding function and/or increased sensory afferent input might be held responsible for the observed increments in alpha-2 power. Thus, further research is required to disentangle the alternative interpretations.

## Data Availability Statement

The raw data supporting the conclusions of this article will be made available by the authors, without undue reservation, to any qualified researcher.

## Ethics Statement

The studies involving human participants were reviewed and approved by Local Ethics Committee of the University of Potsdam (application no. 12/2019). The patients/participants provided their written informed consent to participate in this study.

## Author Contributions

AG, AB, CS, TH, and UG conceived and designed the research. AG and AB conducted the experiments and analyzed the data. All authors contributed to the writing of this manuscript, read, and approved the manuscript.

## Conflict of Interest

The authors declare that the research was conducted in the absence of any commercial or financial relationships that could be construed as a potential conflict of interest.

## Publisher’s Note

All claims expressed in this article are solely those of the authors and do not necessarily represent those of their affiliated organizations, or those of the publisher, the editors and the reviewers. Any product that may be evaluated in this article, or claim that may be made by its manufacturer, is not guaranteed or endorsed by the publisher.
